# Gender Differences in Coping Strategies and Life Satisfaction Following Cognitive-Behavioral and Mindfulness-Based Intervention for Crohn’s Disease: A Randomized Controlled Trial

**DOI:** 10.3390/jcm14051569

**Published:** 2025-02-26

**Authors:** Ganit Goren, Doron Schwartz, Michael Friger, Ruslan Sergienko, Alon Monsonego, Vered Slonim-Nevo, Dan Greenberg, Shmuel Odes, Orly Sarid

**Affiliations:** 1 The Spitzer Department of Social Work, Ben-Gurion University of the Negev, Beer Sheva 8410501, Israel; ganitg@post.bgu.ac.il (G.G.); slonim@bgu.ac.il (V.S.-N.); 2 Department of Gastroenterology and Hepatology, Soroka Medical Center, Beer Sheva 8410501, Israel; doronsh@clalit.org.il; 3 Faculty of Health Sciences, Division of Internal Medicine, Ben-Gurion University of the Negev, Beer Sheva 8410501, Israel; odes@bgu.ac.il; 4 Department of Epidemiology, Biostatistics and Community Health Sciences, The School of Public Health, Faculty of Health Sciences, Ben-Gurion University of the Negev, Beer Sheva 8410501, Israel; friger@bgu.ac.il; 5 The Department of Health Systems Policy and Management, School of Public, Health, Faculty of Health Sciences, Ben-Gurion University of the Negev, Beer Sheva 8410501, Israel; sergienk@bgu.ac.il (R.S.); dangr@bgu.ac.il (D.G.); 6 The Shraga Segal Department of Microbiology, Immunology, and Genetics, Faculty of Health Sciences, The School of Brain Sciences, and Cognition and Regenerative Medicine and Stem Cell Research Center, Ben-Gurion University of the Negev, Beer Sheva 8410501, Israel; alonmon@bgu.ac.il; 7 The National Institute of Biotechnology in the Negev, Ben-Gurion University of the Negev, Beer-Sheva 8410501, Israel; 8 The Guilford Glazer Faculty of Business and Management, Ben-Gurion University of the Negev, Beer Sheva 8410501, Israel

**Keywords:** Crohn’s disease, coping strategies, gender, satisfaction with life, COBMINDEX, cognitive-behavioral mindfulness intervention with daily exercise

## Abstract

**Background and Objective**: Crohn’s Disease (CD) is a chronic inflammatory condition with significant physical and psychological impacts, often requiring comprehensive self-management. This study examines the effects of COBMINDEX (Cognitive–Behavioral and Mindfulness Intervention with Daily Exercise) on coping strategies and life satisfaction in CD patients, focusing on gender-specific responses. Study objectives were to assess the impact of COBMINDEX on adaptive and maladaptive coping strategies and life satisfaction in CD patients, and to examine gender differences in these outcomes. **Materials and Methods**: A pre-planned secondary analysis of a randomized controlled trial, conducted from 2018 to 2021, at two public tertiary hospitals in Israel. A total of 120 CD patients (45 men and 75 women) were randomly assigned to either theCOBMINDEX group or a wait-list control group. Participants were assessed at baseline and post-intervention for coping strategies, mindfulness, psychological symptoms, and life satisfaction using validated scales. Quantile regression explored the gender-specific predictors of life satisfaction. This study was registered at ClinicalTrials.gov (NCT05085925) and Israel Ministry of Health (MOH_2020- 02- 24_008721. asp). **Results**: Both genders showed significant improvements in mindfulness, emotion-focused coping, and active coping (*p* < 0.05). Women exhibited reduced dysfunctional coping and greater emotional support use. For men, emotion-focused coping and mindfulness positively predicted life satisfaction, while for women, reductions in psychological symptoms and dysfunctional coping were significant predictors (*p* < 0.01). **Conclusions**: COBMINDEX enhances coping strategies and life satisfaction in CD patients, with notable gender differences. These findings highlight the importance of gender-tailored psychological interventions to improve overall patient well-being.

## 1. Introduction

This study examines the effects of a **c**ognitive-**b**ehavioral and **min**dfulness-based intervention with **d**aily exercise (COBMINDEX) on adaptive and maladaptive coping strategies in men and women with Crohn’s Disease (CD). Additionally, it explores the relationship between coping strategies and overall life satisfaction following the COBMINDEX intervention.

CD is a chronic inflammatory condition of the gastrointestinal tract, predominantly affecting the distal ileum and colon, with the onset typically occurring in young adulthood [1]. Its prevalence varies across populations; in Europe and the USA, CD affects women approximately twice as often as men, whereas in Asia, men have a higher prevalence [2]. CD is characterized by symptoms such as abdominal pain, diarrhea, weight loss, and anemia, significantly impairing quality of life. Additionally, the disease can lead to severe complications, including bowel obstruction, fistulae, abscesses, and extra-intestinal manifestations [3,4].

Current treatment strategies for CD encompass pharmacological interventions—such as corticosteroids, immunomodulators, biologics, and anti-TNF therapies—alongside dietary modifications and surgical procedures [5,6]. However, these treatments primarily target inflammation and symptom control, rather than address the psychological burden associated with CD [6,7].

### 1.1. The Psychosocial Burden of CD and the Role of Stress

CD extends beyond its physical impact and imposes a significant psychological burden. Patients experience higher stress levels than the general population, which negatively affect disease management and quality of life. Studies consistently show that CD patients face unique psychosocial challenges, including disruptions in daily functioning and interpersonal relationships, contributing to elevated psychological distress [8,9]. A multicenter study revealed that 75% of CD patients identified psychological stress as a factor exacerbating their condition, with those in active disease states reporting even higher levels of stress and poorer mental health outcomes, compared to those in remission [10]. Psychological distress is also associated with increased inflammatory activity, suggesting that stress not only affects mental well-being, but also exacerbates disease progression [11]. This connection highlights the critical need for interventions that address both psychological and physiological aspects of CD management. Recent systematic reviews emphasize that stress contributes to immune dysregulation, exacerbating inflammation and increasing the frequency of disease flare-ups, underscoring the intricate interplay between psychological and physiological factors in CD [12].

### 1.2. Gender Differences in Coping with CD

Significant gender disparities exist in CD, particularly in psychosocial functioning. As many as 65% of women with inflammatory bowel disease (IBD) experience depression and anxiety. Compared to men, they also report higher levels of fatigue, somatic symptoms, eating disorders, and body image dissatisfaction [2,13]. Given this heightened vulnerability, ongoing mental health monitoring and a multidisciplinary approach, integrating mental health professionals, are essential for comprehensive care [13].

While psychological challenges such as depression, anxiety, and sexual dysfunction affect both genders, they tend to be less prevalent and severe in men [2]. However, men with perianal fistulas face unique challenges related to intimacy, sexual dysfunction, and masculinity, highlighting the need for targeted psychosocial interventions [14,15]. Men also generally report better sexual functioning and body image outcomes than women, further emphasizing gender-specific differences in psychosocial concerns [16].

These findings emphasize the importance of gender-sensitive strategies in CD management to ensure tailored psychological and medical support for both men and women.

### 1.3. Addressing Gender Disparities in Research

Gender disparities extend beyond disease burden, to healthcare access and research representation, and have gained increasing attention. One notable issue is the underrepresentation of women in clinical research [17,18,19]. Efforts to address this issue have led to the development of recommendations like the Sex and Gender Equity in Research (SAGER) guidelines, emphasizing the need to incorporate gender perspectives into clinical research [17,19].

However, despite growing awareness, few studies explicitly adopt these guidelines, and research addressing gender differences in IBD management remains limited. Integrating gender considerations into research is crucial for improving treatment personalization, intervention effectiveness, and overall patient outcomes [15].

### 1.4. The Importance of Coping Strategies in CD Management

Given the stress-related nature of CD, there is growing recognition of the importance of self-management programs and stress-reduction interventions. The biopsychosocial model of care, which integrates biological, psychological, and social dimensions, has gained traction as a holistic treatment framework for chronic conditions like CD [20,21,22].

This research underscores the critical role of mental health in influencing disease activity, further supporting the need for interventions targeting both psychological and physiological factors [23,24]. Implementing adaptive coping strategies and self-management techniques can mitigate stress and its impact on disease progression. Effective coping mechanisms have been linked to improved physical function, better psychosocial adjustment, and reduced disease burden [25,26,27]. Furthermore, targeted interventions tailored to individual coping strategies significantly enhance the quality of life by reducing both psychological distress and physiological stressors [28].

For instance, resilience-building interventions, mindfulness training, and cognitive-behavioral techniques have been shown to modulate immune dysregulation, contributing to improved psychological well-being, and to reduce disease flair-ups in the short- and long-term outcomes. These findings emphasize the importance of incorporating stress-reduction techniques into CD management protocols to enhance overall patient well-being [29,30,31,32].

### 1.5. Coping as a Multidimensional Construct

Coping refers to the cognitive and behavioral strategies individuals use to manage the physical and emotional stressors of chronic illness [33]. A classic classification categorizes coping strategies into emotion-focused, problem-focused, and dysfunctional categories [34,35,36]. Emotion-focused coping focuses on reducing emotional distress through strategies such as seeking emotional support, positive reframing, humor, and acceptance. Problem-focused coping directly addresses the stressor through active problem-solving, planning, and seeking instrumental support. Both are considered as adaptive, as they promote effective confrontation and management of illness-related stressors.

Dysfunctional coping refers to strategies that may provide short-term relief but are ultimately counterproductive. These strategies include behavioral disengagement, self-blame, denial, substance use, self-distraction, and venting [34,35,37]. Interestingly, some researchers propose that, under specific conditions, venting negative emotions can have a constructive role. Evidence suggests that venting may aid emotional regulation, enhances resilience, and reduce symptoms of anxiety and depression, particularly when it occurs within supportive environments [38,39]. This perspective reframes venting as a potentially beneficial emotional regulation mechanism, rather than an inherently maladaptive strategy. Further research is needed on venting, as limited studies have examined its role as a coping strategy [40].

Coping strategies and self-esteem are integral to an individual’s psychological framework, shaping how they manage challenges and perceive themselves. While coping strategies tend to be dynamic and context-dependent, self-esteem reflects a more stable evaluation of self-worth across life domains [41,42]. High self-esteem is associated with adaptive coping strategies, such as problem-solving and planning, whereas low self-esteem correlates with maladaptive strategies, including avoidance and self-blame [43].

### 1.6. Mindfulness as an Adaptive Coping Strategy

Mindfulness, defined as the deliberate practice of paying attention to the present moment with a nonjudgmental awareness of one’s thoughts, feelings, and bodily sensations, is an adaptive coping strategy with the potential to buffer stress reactivity [44,45,46]. Mindfulness interventions help mitigate automatic stress responses and foster adaptive coping in individuals with chronic conditions [47]. They redirect attention from negative stimuli, enhancing self-regulation, and reduce maladaptive thought patterns.

Prior studies show that mindfulness-based stress reduction (MBSR) significantly improves psychological well-being by promoting acceptance and reducing symptoms of depression, anxiety, and stress in individuals with chronic pain, even though its impact on attentional patterns is nuanced [48]. Mindfulness training also strengthens both momentary and trait attentional control, supporting emotional regulation by reducing reactivity to negative stimuli [49]. Moreover, mindfulness enhances interoceptive attention, emotional awareness, and resilience, which mitigate negative mood states and improve cognitive adaptability [50].

These findings highlight mindfulness as a valuable tool for stress management and psychological resilience in chronic conditions. By enhancing emotional regulation and reducing stress-related reactivity, mindfulness fosters psychological flexibility, helping individuals develop healthier responses to chronic illness-related stressors.

### 1.7. Coping Patterns in CD Patients

Research on CD patients has explored a wide range of coping strategies [51,52,53,54,55,56,57,58,59,60,61], as well as the role of mindfulness dispositions in shaping psychological well-being [60,62,63,64,65].

Individuals with IBD rely more on maladaptive coping mechanisms than the general population [56]. When comparing CD with ulcerative colitis, CD patients rely more on dysfunctional coping strategies, such as self-blame, behavioral disengagement, and substance abuse, contributing to elevated rates of depression and anxiety [57].

Mindfulness levels are inversely associated with psychological distress; individuals with higher mindfulness scores report lower levels of fatigue, perceived stress, and disease burden [62,63]. Additionally, mindfulness has been identified as a mediator between disease severity and perceived stress, fatigue, and overall health-related quality of life [63]. Variability in coping mechanisms observed across studies may reflect the influence of individual, situational, and disease-related factors [59]. Among these factors, gender plays a critical role in shaping coping patterns, as both biological predispositions and societal expectations contribute to differences in how men and women manage chronic illness.

### 1.8. Gender Differences in Coping Strategies

Research highlights notable gender-based differences in coping strategies, driven by societal and cultural norms that shape traditional psychological roles. Women, when under stress, tend to rely more on emotion-focused coping, such as seeking emotional and instrumental support, compared to men [38]. At the same time, women are also more likely to engage in certain dysfunctional coping strategies, including self-distraction, rumination, self-blame, avoidance, and suppression [38,66]. This dual tendency—greater use of both adaptive and maladaptive coping strategies—illustrates the complexity of gender differences in psychological responses to stress.

Despite these findings, research on gender differences in coping among CD patients remains limited and, at times, inconclusive. Studies suggest that during active disease states, women with IBD report a greater reliance on emotion-focused and problem-focused coping strategies, compared to men [54]. However, in remission, women tend to exhibit a higher reliance on dysfunctional coping mechanisms, in addition to adaptive strategies [55]. Dysfunctional coping strategies, particularly among women, are significant predictors of depressive symptoms [58]. Conversely, emotion-focused coping has been positively associated with higher life satisfaction, particularly in female patients [55].

Another noteworthy observation is that women show greater interest than men in receiving information about psychological disorders from specialists and media sources [54]. Despite these findings, existing research does not provide conclusive evidence regarding gender-specific coping strategies and mindfulness in CD. Additionally, no studies to date have examined how gender influences changes in coping strategies following short-term psychological interventions. This gap in the literature served as a primary motivation for our study. With growing recognition of gender disparities in healthcare, there is an increasing push to integrate gender-specific considerations into clinical research involving IBD patients to improve patient outcomes [15].

### 1.9. Short Term Psyhcological Interventions to Improve Self-Care Managment

The acquisition of adaptive coping strategies is a fundamental goal of cognitive-behavioral interventions [60,67] and mindfulness-based stress reduction interventions [60,62,64,65]. Within these frameworks, patients with CD are trained to identify and alter maladaptive thought patterns, substituting them with more adaptive cognitive processes. Additionally, they learn to modify certain behaviors and employ techniques to manage their pain.

### 1.10. The Concept of Coping Flexibility

Acquisition of coping strategies can be better understood through the lens of coping flexibility, a concept derived from the dual-process theory. Coping flexibility refers to the ability to discontinue ineffective coping strategies and replace them with more adaptive alternatives [68].

This theory underscores the importance of replacing unsuccessful strategies, closely aligning with cognitive flexibility, which involves the ability to shift cognitive frameworks in response to evolving situational demands. The dual-process theory comprised three key stages: (1) re-evaluation, in which individuals assess the effectiveness of their current coping strategies; (2) abandonment, where ineffective strategies are discarded; and (3) re-coping, where more adaptive strategies are adopted. This dynamic and iterative approach allows individuals to refine their coping mechanisms in response to the changing stressors.

Empirical evidence suggests that a psychological flexibility helps mitigate psychological distress by reducing avoidant coping and by fostering approach-oriented coping strategies [69].

### 1.11. Coping Flexibility and Gender

Recent research suggests that men tend to have a less flexible gender schema than women, which may impact their ability to adapt to various situations [70]. Theoretical and empirical evidence suggest that adherence to traditional masculinity negatively impacts men’s stress severity and treatment engagement [71]. This gender-based disparity in coping flexibility could impact how men and women respond to psychological interventions.

In contrast, women often navigate multiple life domains, including caregiving, professional responsibilities, and societal expectations [72]. These multifaceted roles require substantial coping flexibility, which may explain why women exhibit greater adaptability in their coping mechanisms. Given this broader societal and caregiving involvement, women may be more receptive to psychological interventions like COBMINDEX, incorporating new coping strategies more effectively than men [73,74].

Men, on the other hand, may demonstrate lower coping flexibility due to societal pressures discouraging emotional expression and perceived vulnerability [16]. Research indicates that women’s greater coping flexibility enables them to adopt new strategies more readily, making gender-sensitive interventions particularly relevant [73,74].

### 1.12. The COBMINDEX Program: A Targeted Intervention

An example of an intervention integrating these principles is COBMINDEX, a short-term psychological framework specifically tailored for CD patients [60]. This comprehensive intervention combines cognitive-behavioral techniques, mindfulness-based stress reduction strategies, and daily self-exercises of the techniques learned, to holistically address both psychological and physiological aspects of disease management.

Throughout this process, participants develop essential coping skills, including the reduction in maladaptive strategies such as self-blame, social withdrawal, and denial, while fostering adaptive strategies like acceptance, self-compassion, proactive engagement, and effective tools for managing pain and suffering. This structured self-exercise component reinforces skill acquisition, encourages adherence, and promotes long-term integration of adaptive coping mechanisms, ultimately enhancing emotional regulation and improving the management of psychological distress and disease burden [75,76,77].

By modifying maladaptive thought patterns, enhancing emotional regulation, and fostering physical well-being, COBMINDEX aims to alleviate psychological distress, promote adaptive coping strategies, and improve the overall quality of life.

A review of randomized controlled trials (RCTs) conducted in the past five years highlights that most psychological interventions for CD patients focus either on cognitive-behavioral or mindfulness-based strategies, to reduce distress and improve well-being [65,78,79,80,81,82]. COBMINDEX, however, distinguishes itself by uniquely integrating these approaches into a single intervention. This comprehensive structure offers a diverse range of stress-reduction techniques, allowing patients to choose strategies that align with their personal preferences and needs.

Furthermore, COBMINDEX leverages one-on-one video conference sessions with a trained social worker, ensuring personalized support and tailored guidance. This innovative use of virtual sessions not only expands access to therapy for patients who live far from clinical facilities, but also enhances the quality and flexibility of care delivery. By combining its unique structure and accessibility, COBMINDEX represents a comprehensive and adaptable approach to psychological care for IBD patients.

A groundbreaking aspect of COBMINDEX, as highlighted in the study by Riggoee et al. [83] while evaluating effects of psychological therapy on CD patients, is that unlike other interventions that focus primarily on patients in remission, COBMINDEX has demonstrated significant benefits for individuals actively experiencing disease symptoms. This includes reductions in disease activity, psychological distress, and fatigue, showcasing the program’s capacity to address the multifaceted challenges faced by patients with active CD. This focus on clinically active CD makes COBMINDEX particularly relevant and impactful, addressing a critical gap in psychological care for this patient population.

### 1.13. Primary vs. Secondary Analyses of COBMINDEX: Disease Outcomes, Psychological Effects, and Novel Insights

The primary analysis of the COBMINDEX intervention, conducted by Goren et al. [60], focused on improving disease-related quality of life among CD patients. The analysis also demonstrated reductions in disease activity, fatigue, and psychological symptoms, highlighting the intervention’s significant impact on patients’ well-being.

Additionally, various secondary analyses were conducted to explore the further dimensions of COBMINDEX’s effectiveness. Among these, Regev et al. [84] found that COBMINDEX enhances life satisfaction through reductions in perceived stress and interpersonal sensitivity, identifying these psychological factors as critical drivers of the intervention’s impact. Moreover, additional secondary analyses by Regev et al. [85] demonstrated that the intervention significantly alleviated abdominal pain and fatigue, which contributed to improvements in work productivity and daily functioning.

By combining the biological findings with the psychological outcomes of the COBMINDEX intervention, a secondary analysis by Nemirovsky et al. [32] demonstrated a strong correlation between lower baseline levels of inflammatory biomarkers and greater reductions in disease symptoms. Similarly, lower baseline cortisol levels, a key stress-related hormone, were associated with more substantial improvements in mental health outcomes. Furthermore, Ilan et al. [86] reported significant changes in microbial indices after COBMINDEX, with some of these changes linked to psychological manifestations and systemic inflammation, indicating the intervention’s potential role in mitigating the pathobiology of CD.

### 1.14. Gender Considerations in COBMINDEX Outcomes

In the current article, a secondary analysis investigates the role of gender in moderating the effectiveness of COBMINDEX. Specifically, it examines whether gender influences the patients’ capacity to benefit from the intervention, and how these differences affect changes in life satisfaction among CD patients. By focusing on gender as a potential moderator, the secondary analysis builds on the primary findings of the original trial, aiming to provide a more detailed understanding of how demographic factors, such as gender, may shape the intervention’s impact on specific patient groups.

### 1.15. Study Objectives

This study aims to examine the impact of COBMINDEX on gender-specific differences in coping strategies and life satisfaction among CD patients.

The first objective is to compare the impact of COBMINDEX on adaptive and maladaptive coping strategies among men and women with CD. We hypothesize that women will develop a broader range of coping strategies than men after participating in COBMINDEX. This prediction is based on well-established gender differences in coping mechanisms, where women tend to engage in emotion-focused coping and social support-seeking more frequently than men [38,54,55]. Additionally, research suggests that women exhibit greater coping flexibility, which allows them to adopt new strategies more easily when exposed to psychological interventions [69,72,73]. COBMINDEX, which integrates mindfulness, emotional regulation, and cognitive reframing, aligns well with women’s natural coping tendencies. As a result, we expect that women may be more inclined than men to incorporate a broader set of coping strategies following the intervention.

The second objective is to assess the extent to which changes in coping strategies influence the overall life satisfaction, and whether these changes differ by gender. We assume that coping strategies will enhance life satisfaction significantly more in women than in men. Research has demonstrated that coping strategies are strong predictors of well-being, with adaptive coping linked to better psychological outcomes and dysfunctional coping associated with increased distress [34,35,36,43,58]. However, gender differences exist in the specific pathways through which coping strategies influence life satisfaction. For women, reductions in psychological distress and dysfunctional coping appear to be the strongest predictors of improved well-being [55]. In contrast, men’s life satisfaction is more closely linked to emotion-focused coping and mindfulness [55]. Since coping flexibility enhances psychological resilience and emotional regulation, women—who generally exhibit greater coping flexibility—may experience stronger improvements in life satisfaction. This aligns with the previous findings, suggesting that reductions in psychological distress and dysfunctional coping contribute to well-being more significantly in women than in men.

By incorporating gender considerations into this analysis, this study aims to provide a more nuanced understanding of the psychological benefits of COBMINDEX in CD patients. Examining these gender-specific effects will not only improve our understanding of how men and women respond differently to psychological interventions, but also inform the development of targeted, gender-sensitive interventions, to enhance coping and overall well-being in individuals with CD.

## 2. Materials and Methods

The current study is a pre-planned secondary analysis of a randomized controlled parallel-group superiority COBMINDEX interventional trial with a 1:1 allocation ratio, among CD patients [60]. The study was performed from July 2018 to February 2021, at two public tertiary hospitals that have been conducting a series of clinical and basic science research projects in CD patients for ten years, as part of the Israeli IBD Research Nucleus. The study sites included Soroka Medical Center, Beer-Sheva, and Rabin Medical Center, Petah Tikva, Israel. Participating gastroenterologists were all board-certified; participating clinical social workers were all required to attend a special course in the study methods and tools; the other participants were university-level clinical researchers and statisticians. The study was conducted in accordance with the Declaration of Helsinki. Prior to study commencement, approval was obtained from the Ethics Committee at Soroka Medical Center, Beer Sheva (Approval numbers: 0156-17-SOR and 0063-20-SOR), and the Ethics Committee at Rabin Medical Center, Petah Tikva (Approval number: 0788-18-RMC). All participants received a detailed explanation of the study’s purpose, procedures, potential risks, and benefits. Written informed consent was secured from every participant by a study physician involved in the study. Patient-identifying data were kept separate from the study database. Only the specific social worker allocated per patient had access to that patient’s name and telephone number.

### 2.1. Participants

Patients aged ≥18 years, with mild-to-moderate CD (range 5–16), according to the Harvey-Bradshaw Index (HBI) [87,88,89], were recruited by advertising at participating hospitals, the Israel Foundation for Crohn’s Disease and Ulcerative Colitis website, and social networks. Exclusion criteria included a diagnosis made within the past 12 months, initiation of new medication within the last three months, surgery performed in the past six months, planned surgical procedures, pregnancy, current or past psychiatric disorders or psychiatric medication use, irritable bowel syndrome, and insufficient proficiency in reading and speaking Hebrew, as meetings with social workers and questionnaires were conducted in Hebrew [60]. Participation in a concurrent trial was not allowed. Patients were also excluded from the trial due to lack of interest, failure to complete required assessments (questionnaires or blood samples), onset of pregnancy, or the need for surgery. Change in medication was not a criterion for stopping a patient’s participation. Two clinical social workers performed the initial screening of participants, and two expert gastroenterologists reviewed the screening procedures, confirmed the diagnosis, calculated the HBI, and enrolled eligible patients, who then provided their signatures on consent forms. Patients continued their regular medical follow-ups with their private physicians, irrespective of their participation in the study. Except for the gastroenterologists and social workers, the patients’ identities were unknown to the research personnel. A sample size calculation based on α = 0.05, power = 0.8, and SD = 14 indicated the need to enroll ≥86 patients.

#### 2.1.1. Baseline Characteristics of Study Participants

The final study sample included 120 participants, with 45 men (37.5%) and 75 women (62.5%). Participants were similar in terms of age, socioeconomic status, education level, employment, and smoking habits. However, men had a longer median disease duration compared to women. Table 1 presents the demographic and clinical characteristics of the study cohort.

#### 2.1.2. Participant Enrollment and Randomization

**Figure 1:**
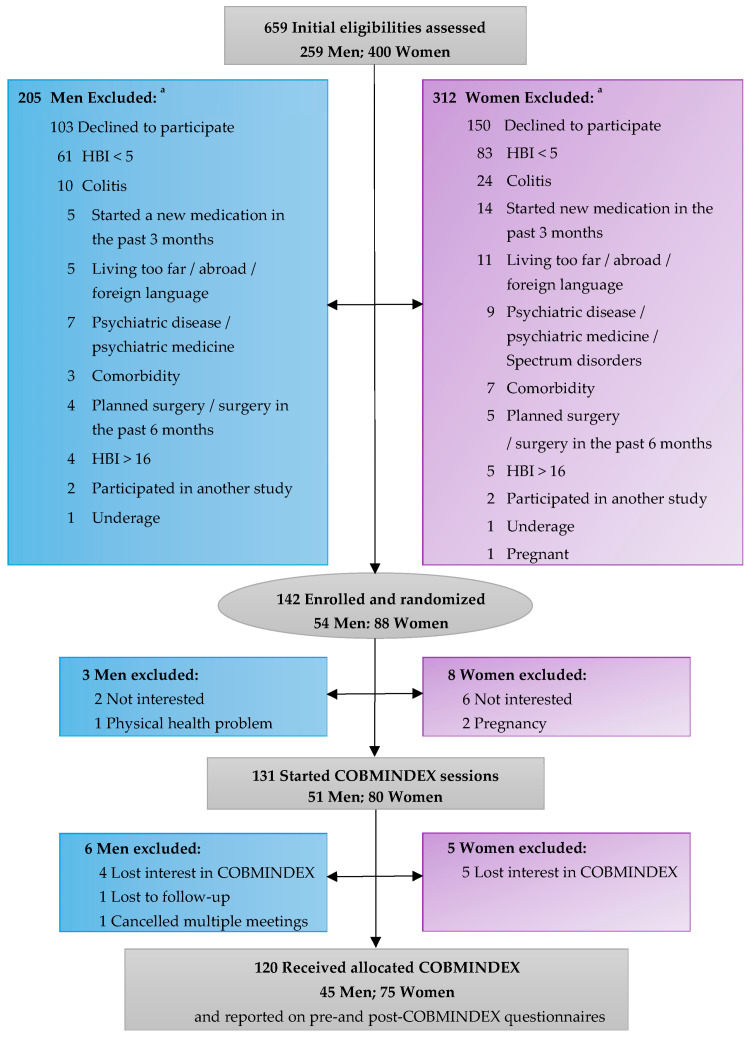
Consort diagram of participants in the study. HBI, Harvey Bradshaw Index. ^a^ Individuals could meet more than 1 exclusion criterion.

A total of 659 patients expressed interest in the study; out of these 142 met the eligibility criteria and were enrolled. These participants were then randomly assigned to one of two groups: the COBMINDEX group, which received training over three months, or the wait-list control group, which was scheduled to receive the COBMINDEX training after an initial three-month waiting period (those data are not part of this report). During the study, 22 participants withdrew from the trial, leaving 120 patients who successfully completed the trial. Of the 22 participants who dropped out, 3 men were excluded before starting COBMINDEX sessions: 2 were not interested, and 1 was excluded due to an HBI score that exceeded the allowable threshold. Similarly, 8 women were excluded before starting the sessions: 6 were not interested, and 2 were excluded due to pregnancy. During the COBMINDEX sessions, 6 men lost interest and discontinued participation after completing 2–6 sessions, 1 man completed 7 sessions but did not follow-up, and 1 man was excluded after three instances of last-minute cancelations of sessions with a social worker. Additionally, 5 women discontinued participation during the COBMINDEX sessions due to loss of interest. The data presented herein reflect the outcomes and analyses pertaining to these 120 participants (refer to Figure 1 for a consort diagram of participants in this study).

### 2.2. Patients Procedures

All participants completed the baseline medical assessments and online socio-demographic and psychological questionnaires at study entry. They self-assessed their economic status on a scale of 1 (not good at all) to 5 (very good). Each participant also provided a blood sample for the measurement of C-reactive protein (CRP), a marker of inflammation, with 0.5 mg/dL set as the upper normal limit. Subsequently, patients were randomly assigned to either the COBMINDEX group or the wait-list control. After three months, another medical assessment was conducted, online questionnaires were readministered, and participants provided additional blood samples for CRP levels. Study questionnaires were administered using REDCap (Research Electronic Data Capture), a secure and validated platform for online data collection, developed by Vanderbilt University (Nashville, TN, USA).

### 2.3. Randomization

The randomization was conducted using computer-generated cluster random sampling method with proportionate allocation by gender. The randomization sequence was generated by an independent coordinator with no clinical interaction with participants. Study coordinators, responsible for participant recruitment, enrolled participants and assigned them to study groups based on the randomization sequence provided by the independent coordinator. This process ensured that the allocation was performed consistently and without bias, minimizing potential risks to the validity of outcome assessments. No restrictions, such as blocking or stratification, were applied. Unblinding was not allowed under any circumstances.

Baseline assessments, including clinical evaluations and blood sample collection, were conducted prior to the randomization to ensure blinding at this stage. Blood samples were labeled only with patient identification numbers and sent to the laboratory without any indication of group allocation. Randomization occurred only after these assessments were completed, ensuring that neither gastroenterologists nor the laboratory staff were aware of group assignments during the baseline phase.

After randomization, participants were informed of their group allocation. Due to the nature of the study design, participants in the intervention group actively engaged in sessions with a social worker, while participants in the wait-list control group received no intervention during the study period. This unavoidable difference in participant behavior made it impractical to blind participants to their group assignment.

For post-intervention assessments, measures were implemented to maintain blinding at the level of the assessors, including gastroenterologists and laboratory staff. Patient files provided to gastroenterologists for follow-up assessments contained no information about group allocation. Laboratory staff received only blood samples labeled with patient identification numbers, with no reference to group assignment. Furthermore, the COBMINDEX intervention did not leave any visible clinical markers that could inadvertently reveal group assignment. These measures minimized the likelihood of bias in outcome assessments.

However, no formal blinding test, such as asking assessors to guess group allocation, was conducted. This decision was based on logistical considerations, as the absence of clinical or behavioral indicators that could compromise assessor blinding was deemed sufficient to ensure masking. Nonetheless, the lack of formal testing remains a limitation of the study.

Participants in both the intervention and wait-list control groups completed assessments at identical time points, using the same protocols. The wait-list control group was informed they would receive the same COBMINDEX intervention as the intervention group, including identical content and duration, after the completion of the study period.

### 2.4. Patient Retention Measures

Clinical visits were scheduled by the study coordinator, who also managed participant follow-up and ensured timely data collection. Participants in the intervention group attended two visits: one at baseline and another after completing the COBMINDEX program (three months later). Participants in the wait-list control group attended three visits: one at baseline, one after three months on the wait-list (pre-COBMINDEX), and a final visit after completing the delayed intervention (six months from baseline). Gastroenterologists conducted all clinical visits at predefined time points.

To improve adherence, visits were scheduled on dedicated “study days” to accommodate the patients’ schedules. If a patient missed a visit without prior notice, the study coordinator sent a reminder via smartphone message. If no response was received within 24 h, a follow-up phone call was made. Missed visits requiring rescheduling were rare.

For online sessions with the social worker, patients received reminders via smartphone if they missed a session. After a second missed session, the intervention’s psychological manager contacted the patient to assess motivation and discuss participation. Patients were informed that three missed sessions would result in removal from the intervention. Ultimately, only one patient was removed from the study after missing three sessions, in accordance with the retention protocol.

### 2.5. Protocol Modifications

Requests for protocol modifications were to be addressed immediately to the ethics committees at the participating hospitals. However, no such need arose during the trial.

### 2.6. Harms

It was not anticipated that any harm would occur to any participant. Therefore, no specific provision was made for this, but each case would be handled ad hoc if required.

### 2.7. Dissemination of the Study Results

The study results will be disseminated through presentation at patient meetings, in the media, and by publication in medical literature. The investigators have committed to personally handling the dissemination of the study results without the assistance of a professional writer. All investigators will be included in any scientific publications. The dataset will not be made available to a third party in compliance with the ethical regulations.

### 2.8. Biological Specimens

This study incorporated the taking of biological specimens (other than blood for CRP); these were used to generate data reported elsewhere [32,86].

### 2.9. COBMINDEX Program Content

The COBMINDEX program follows a gradual and structured learning approach. It begins with foundational behavioral techniques including abdominal breathing, body scanning, and progressive muscle relaxation. It then progresses to guided imagery techniques, identification of negative core beliefs and development of more positive, adaptive, and realistic ones. The program culminates in mindfulness meditation practices which foster awareness of thoughts, feelings, and unpleasant sensations, as well as promote compassion for oneself and others.

Patients were instructed to practice the learned techniques daily, for at least ten minutes, using guided audio podcasts. They self-reported their practice via a smartphone link specifying which techniques they used and how frequently they used them. Further details regarding self-practice are available in Regev et al. [84].

### 2.10. COBMINDEX Intervention Delivery

Licensed clinical social workers delivered the COBMINDEX program over three months, through seven consecutive one-on-one video conference sessions (Skype^TM^ 8.x, Microsoft Corporation, Redmond, WA, USA), each lasting at least one hour. These sessions followed a structured and comprehensive manual and incorporated various psychological and relaxation techniques, as documented by Goren et al. [60,84].

### 2.11. Questionnaires

The study employed validated tools to measure disease activity (Harvey-Bradshaw Index), coping strategies (Brief COPE inventory), mindfulness (Freiburg Mindfulness Inventory), psychological symptoms (Global Severity Index) and life satisfaction (Satisfaction with Life Scale).

The Harvey-Bradshaw Index (HBI) [87] is a CD-specific questionnaire evaluating disease activity regarding symptoms, abdominal mass, and complications. Disease activity is defined as remission (<5), mild (5–7), moderate (8–16), or severe (>16). The HBI was selected as a simple and validated tool for assessing CD activity, which is critical for determining disease severity. Its continued use in recent research highlights its relevance for clinical and research applications [88,89]. It remains one of the most widely used non-invasive indices for monitoring CD activity, offering both clinical utility and research reliability. We used a validated Hebrew version of the scale [90].

The Brief Coping Operations Preference Enquiry (Brief COPE) [34] is a comprehensive tool for assessing coping mechanisms across 28 dimensions, each rated on a four-point scale. The summation of these items’ scores results in 14 distinct coping subscales, which are further organized into three primary coping strategy categories. A higher cumulative score within any category suggests a greater reliance on that coping strategy. The categorization of strategies encompasses emotion-focused coping, entailing the use of emotional support, positive reframing, humor, acceptance, and religion strategies; problem-focused coping, which involves active coping, seeking instrumental support, and engaging in planning; and dysfunctional coping, characterized by self-distraction, denial, substance abuse, behavioral disengagement, venting, and self-blame. In alignment with the methodology of Baumstarck et al. [39,91], our analysis recalibrates the dysfunctional coping score by omitting the venting subscale. The Brief COPE was chosen for its ability to differentiate between adaptive (problem-focused and emotion-focused coping) and maladaptive (dysfunctional coping) mechanisms, which are crucial for understanding how patients with CD manage chronic illness-related stress. Its application in chronic illness populations, including inflammatory bowel disease, ensures its relevance in assessing psychological resilience and adaptation strategies [92]. We used a validated version of the scale in Hebrew [93].

The Freiburg Mindfulness Inventory (FMI) [94] is a 14-item questionnaire. Each item can be answered on a scale between 1 and 4. The scores were summed up and ranged from 14 to 56. A higher score indicates a greater mindfulness level. The FMI was chosen due to its ability to assess mindfulness as a trait, rather than as a temporary state, making it suitable for evaluating the effects of long-term mindfulness-based interventions. Research indicates that mindfulness plays a critical role in stress regulation and emotional well-being in chronic disease populations, including CD patients. The FMI translated to Hebrew was used in other published studies [95].

The Brief Symptom Inventory (BSI) [96] is a 53-item questionnaire. It assesses nine psychological symptoms experienced in the past month (depression, somatization, obsession-compulsive, interpersonal sensitivity, anxiety, hostility, phobic anxiety, paranoid ideation, and psychoticism). A higher score implies more severe symptoms. The BSI yields a summary score, the Global Severity Index (GSI), with a range of 0–4. The BSI was chosen for its ability to assess a broad range of psychological symptoms relevant to CD patients. The validated Global Severity Index (GSI) provides a reliable measure of overall psychological burden, making it a suitable tool for evaluating mental health in chronic illness populations. Its validity and reliability have been widely confirmed in both clinical and community settings [97]. We used the validated Hebrew translated version [98].

The Satisfaction with Life Scale (SWLS) [99] is a 5-item instrument designed to measure global cognitive judgments of satisfaction with one’s life. The score range is 5–35; a higher score indicates more satisfaction with life. The SWLS was selected due to its strong psychometric properties, including a high internal consistency and validity across diverse populations. Life satisfaction is a crucial determinant of well-being in individuals with chronic diseases, and this scale provides a standardized measure widely used in health and psychological research. Its robustness allows for cross-population comparisons, reinforcing its applicability in assessing quality of life in CD patients. It is used in many recently published studies [100]. We used a validated translated to Hebrew version of the scale [101].

### 2.12. Outcome Measures

d: The primary outcomes of the study were the changes in *coping strategies* among CD patients following the COBMINDEX invention. These were assessed using the Brief COPE Inventory.

Secondary Outcome Measures: The secondary outcomes reflect the broader psychological and physiological effects of the primary outcomes and include changes in:

Satisfaction with life, assessed using the Satisfaction with life Scale (SWLS); mindfulness levels, assessed via the Freiburg Mindfulness Inventory (FMI); psychological symptoms, evaluated through the Global Severity Index (GSI) which provided a composite score of psychological distress and disease activity and inflammation, assessed via the Harvey-Bradshaw Index (HBI) for symptom severity and C-reactive protein (CRP) levels for inflammation.

### 2.13. Assessment Schedule

Baseline assessments included demographic, clinical and psychological evaluations using the tools described above, along with blood sampling for CRP. Questionnaires were completed online via a secure platform, while clinical visits were conducted for blood sample collection and physician-administered evaluations of disease activity using the Harvey-Bradshaw Index (HBI).

Post-intervention assessments were conducted three months after baseline, following the completion of the COBMINDEX intervention. The same procedures were followed: online completion of questionnaires and clinical visits for blood sampling and HBI evaluations. This approach ensured consistency and reliability across assessment points.

No changes to trial outcomes were made after the trial commenced.

### 2.14. Statistical Analysis

The dataset was accessible only to the study statisticians, in accordance with the ethics committee’s regulations.

Data were analyzed using IBM SPSS Statistics 26 for Windows (IBM Corp: Armonk, NY). This SPSS version was used by other recently published social science studies [102]. Statistical significance was set at *p* < 0.05. Categorical (qualitative) variables were summarized as frequencies and percentages. The quantitative variables were not normally distributed and are presented as a median and interquartile range [IQR]. Economic status is shown as a dichotomic variable (low or middle-high) since most patients’ scores on economic status indicated middle or high status.

Gender differences in data pre- and post COBMINDEX were appraised by Pearson’s χ^2^ test for qualitative variables and Mann–Whitney U test for quantitative variables.

Change between pre-and post-COBMINDEX data is presented as a relative change. For detailed information, please see Goren et al. [60].

Since the satisfaction with life score was not normally distributed, we performed quantile regression for percentiles 10%, 25%, 50%, 75%, and 90%, separately for men and women. Quantile regression is used in non-normally distributed data analysis when there is an interest in group differences across the distribution of a given dependent variable, rather than only in the mean. The relationship between the predictor and the dependent variable is non-linear, and we wanted to examine the slopes of the regression line that vary across the quantiles of the dependent variable. Quantile regression modeling is widely used in psychology research to analyze non-normally distributed data [103,104,105].

## 3. Results

### 3.1. Compliance with Protocol

All patients completed the scheduled online sessions with the social workers. Among women, the percentages of those exercising ≥10 min/day over the 3-month period for up to 25%, 26–50%, 51–75%, and >75% of days were 5.4%, 16.2%, 23.0%, and 55.4%, respectively. For men, the respective percentages were 6.8%, 18.2%, 20.5%, and 54.5%. No significant difference was found in daily exercise rates between men and women (*p* = 0.971).

### 3.2. Effect of COBMINDEX on Psychological Measures Between and Across Genders

Men and women had comparable pre-COBMINDEX values for HBI, CRP, satisfaction with life, and GSI (Table 2a). Men and women showed comparable pre-COBMINDEX values for mindfulness and the three forms of coping, except for women reporting higher instrumental support use than men (4 vs. 5, *p* < 0.044) (Table 2b).

In the between-subject analysis, following COBMINDEX, women used more emotional support (5 vs. 6, *p* = 0.001) and more instrumental support than men (5 vs. 6, *p* = 0.010). Furthermore, only women exhibited a significant decrease in dysfunctional coping (19 vs. 18, *p* < 0.024) (Table 2b).

Table 2a,b also show the changes due to COBMINDEX within each gender. Both genders reported enhanced life satisfaction and reduced psychological symptoms as measured by GSI (Table 2a). As far as coping concerns, men and women demonstrated a significant increase in the use of mindfulness, emotion-focused coping, such as positive reframing and religion, and the subscale of active coping (part of problem-focused coping). Only women reported a significant increase in emotional support subscale (*p* = 0.001). In problem-solving coping, only women reported an improvement (*p* = 0.014). In dysfunctional coping, only women used less dysfunctional coping (*p* = 0.016), specifically, less behavioral disengagement (*p* = 0.011) and less self-blame (*p* = 0.003). Please note the changes in the range and IQR in Table 2b.

### 3.3. Determinants of Life Satisfaction Among Men and Women with CD

Table 3 presents a quantile regression model of determinants of satisfaction with life, separately for men and women with CD. The calculation was made using the relative change (rΔ) for each dependent and independent variable. Emotion-focused coping was a significant positive predictor of life satisfaction at the 10th percentile in men (β = 0.44, *p* < 0.001) but was not a significant predictor in women. Dysfunctional coping negatively predicted life satisfaction in men in the 10th (β = −0.57, *p* < 0.001) and 25th (β = −0.65, *p* < 0.05) percentiles, and in women in the 50th (β = −0.36, *p* < 0.05) and 75th (β = −0.81, *p* < 0.05) percentiles. Mindfulness was a positive predictor of satisfaction with life in men in the 25th (β = 0.51, *p* < 0.05) and 50th (β = 0.53, *p* < 0.05) percentiles. GSI, which measures psychological symptoms, was a negative predictor among women in the 10th, 25th, and 50th percentiles (*p* < 0.01; Table 3).

### 3.4. Harms and Adverse Events

No adverse events or unintended effects were reported by participants in either the intervention or wait-list control groups, throughout the study period. Given its design and application, the COBMINDEX intervention was deemed safe before the study, with no anticipated harm. The absence of adverse events supports the safety and feasibility of the intervention in this population.

## 4. Discussion

### 4.1. Gender Comparisons in Coping and Psychological Measures

In this gender-specific analysis, men and women with CD showed comparable baseline scores for CD symptom severity (HBI), CRP levels, life satisfaction, and psychological symptoms (GSI) before the COBMINDEX intervention. Baseline assessments revealed no significant gender differences in mindfulness utilization or coping strategies, except for instrumental support—an aspect of problem-focused coping—which women used more frequently. These findings align with prior research, suggesting minimal gender-based differences in the preference for adaptive versus maladaptive coping strategies [106,107]. However, they contradict other studies that have identified gender-specific variations in these domains [108].

### 4.2. Impact of COBMINDEX on Coping Strategies

We anticipated that following the COBMINDEX intervention, women would develop a wider range of coping strategies than men due to their greater gender schema flexibility. Interestingly, both men and women showed significant improvements in mindfulness utilization and emotion-focused coping strategies, including positive reframing, religion, and the active coping (a subscale of problem-focused coping). However, a key gender difference emerged: women also reported a significant increase in emotional support-seeking, which was not observed in men. Furthermore, only women exhibited improvements in problem-solving coping, along with a notable reduction in dysfunctional coping—particularly in behavioral disengagement and self-blame.

### 4.3. Potential Explanations for Gender Differences

One possible explanation for the observed gender differences following the COBMINDEX intervention is adherence to self-practice within the study protocol. However, as men and women attended the teaching sessions and engaged in the assigned exercises at similar rates, other factors likely contributed to the observed disparities. One plausible factor is the format of the intervention itself, which may have resonated more strongly with women. Previous interventional studies on patients with substance use disorders have similarly found that women tend to benefit more from short-term psychological interventions than men [109].

The theoretical framework proposed by Reskin and Bielby [110] provides further insight into these gender differences, emphasizing how societal expectations shape coping strategies [111]. Women’s traditional roles, which often combine professional and caregiving responsibilities, create a dual burden that necessitates greater coping flexibility. In this context, the COBMINDEX intervention may have acted as a catalyst for fostering more adaptive coping mechanisms, explaining the significant reduction in self-blame and behavioral disengagement observed among women. These shifts suggest a dynamic adjustment in their coping strategies to effectively manage both their disease and the multiple responsibilities they juggle.

Moreover, these gender-based variations in coping flexibility may be attributed to the greater gender schema flexibility that women tend to possess, which enables them to more readily adopt and implement new coping mechanisms [70]. This tendency to continuously refine coping abilities is reinforced by the need to navigate multiple social roles, including career responsibilities, child-rearing, and spousal relationships. Unlike men, whose gender roles have traditionally been more rigidly defined, women must constantly adapt to diverse and sometimes conflicting demands. As a result, they actively seek psychological education and mental healthcare services more frequently than men, reinforcing a cycle of self-improvement and coping expansion [13].

Conversely, men’s traditional roles have historically emphasized economic provision, with fewer expectations for emotional engagement and caregiving. This relative stability in gender role expectations may explain why men’s coping mechanisms have remained more consistent over time. However, gender roles are gradually evolving, reflecting shifts in societal attitudes toward masculinity and caregiving. Despite these changes, coping flexibility remains a more pressing issue for women, as their roles have historically required greater adaptation to a wider range of social and professional expectations.

COBMINDEX, which integrates cognitive-behavioral and mindfulness-based techniques, targets thought processes, emotional regulation, and behavioral adaptation. Mindfulness practice, in particular, enhances self-regulation, attention control, and emotional awareness, forming a key component of the transtheoretical model [112]. The gender differences observed in emotional support-seeking and emotion-focused coping strategies may be partially explained by the tendency for women to be socialized toward a greater emotional expressiveness and interpersonal support-seeking behaviors [113]. Attending COBMINDEX may have heightened these capacities in women, potentially counteracting the emotional suppression often seen in those managing chronic illness. Furthermore, the specific content of the intervention—which may have resonated more strongly with challenges commonly faced by women—could explain why their emotional responses were more pronounced than those of men.

Our second objective was to assess how the changes in coping strategies influence the life satisfaction. We hypothesized that these coping strategies would have a greater impact on improving women’s life satisfaction, rather than men’s. However, the regression analysis revealed that positive associations between changes in mindfulness, coping strategies, and increased life satisfaction were observed exclusively in men.

This phenomenon can be understood through several perspectives. One explanation lies in societal expectations and gender roles, where men are often encouraged to adopt a “bear and grin” approach, emphasizing stoicism and task-oriented coping. Mindfulness, as an inward-focused practice, aligns with these norms, providing men with a socially acceptable mechanism to regulate stress while maintaining traditional masculine ideals [71,114,115]. Unlike women, who tend to use a broader spectrum of coping strategies—including external support and emotional expression—men may experience greater benefits from mindfulness practices as a means of stress regulation. This internal management of emotions and stress, facilitated by mindfulness, aligns with the problem-solving, task-oriented management, typically associated with male roles. Therefore, while mindfulness benefits both genders, its impact on enhancing life satisfaction is apparently more pronounced in men.

The regression analysis indicated that the Global Severity Index (GSI), a measure of psychological stress, negatively correlated with life satisfaction in women, suggesting that as the stress decreased, life satisfaction improved. However, no such association was found in men. Several psychological, societal, and methodological factors may explain this outcome.

The societal gender roles of women may offer a plausible explanation for this result. Traditionally, a woman’s caregiving role can lead to increased anxiety, depression, and stress. As women learn new strategies through COBMINDEX, their capacity for emotional regulation and cognitive control could improve, helping to mitigate undesirable emotional responses [116,117]. This enhancement in cognitive flexibility might, in turn, lead to changes in life satisfaction. While women are more likely to acknowledge and articulate their emotional struggles, making their perceived reductions in stress more likely to translate into reported improvements in life satisfaction, men are often socialized to internalize stress, and may not consciously perceive psychological distress as a factor influencing their overall well-being. As a result, even if men experienced stress reduction through COBMINDEX, this improvement may not have been reflected in their self-reported life satisfaction. Moreover, masculine norms discourage emotional vulnerability, emphasizing self-reliance and resilience [71]. Studies show that men often hesitate to engage with psychological interventions fully or to view emotional regulation as integral to their quality of life [118]. This reluctance to emotionally engage with interventions may weaken the link between stress reduction and overall satisfaction.

In this trial, neither men nor women exhibited an expansion in their range of coping strategies, nor did they experience a notable increase in life satisfaction. This observation could be interpreted through the lens of the neurovisceral integration model, which posits that the development of emotional regulation and problem-solving practices, along with the reduction in dysfunctional strategies, occurs as a synergistic process. The model emphasizes cognitive control, inhibiting unwanted responses and enhancing cognitive flexibility [105]. The elimination of dysfunctional habits is crucial, as it positions individuals to regulate their reactions to chronic illness better. This step might be fundamental in triggering changes in other coping domains.

It is possible that learning and practicing COBMINDEX may require a longer duration to internalize a multitude of emotion-focused and problem-focused coping skills. As noted, women improved their emotion and problem-solving skills, but no effect was detected on satisfaction with life. It is suggested that women require more extended periods to integrate problem-focused and emotion-focused strategies to improve their satisfaction with life. However, the situation for men may be more complex and involve psychological components that were not measured in the current study. In contrast with women, men traditionally are less likely to seek medical assistance and treatment for IBD treatment [119] as well as mental health issues [120,121]. Among young men, significant barriers include stigmatizing beliefs, challenges in identifying or expressing concerns, a preference for self-reliance, and difficulties accessing help [114,122]. As a result, men who engage in psychological interventions may initially approach them with skepticism or negative attitudes and hold lower expectations [123].

### 4.4. Study Limitations

While this study provides valuable insights into gender differences in coping strategies among CD patients, several limitations must be acknowledged.

First, the cohort comprised 45 men and 75 women, resulting in an unequal gender distribution that may affect the generalizability of the findings. Although no significant gender differences were observed in age, socio-demographics, employment status, smoking habits, medications, or disease phenotype (Table 1), men had a longer disease duration than women. This difference could influence coping mechanisms and life satisfaction independently of gender. However, previous research indicates that women often experience diagnostic delays due to prolonged evaluations and a higher frequency of misdiagnoses across various levels of the healthcare system [124]. This suggests that women may contend with CD for longer durations than documented, despite men in our cohort exhibiting longer disease durations.

Second, the smaller sample of male participants may have limited our ability to detect broader trends in their coping strategies. However, despite this limitation, the observed gender differences were statistically significant, reinforcing the reliability of our findings. Nevertheless, the gender imbalance may have reduced our capacity to detect more subtle coping patterns in men. This limitation may have obscured potential associations between psychological stress, coping mechanisms, and life satisfaction among men. Future studies with a more balanced gender distribution will be essential to enhance the generalizability of results and provide a more comprehensive understanding of gender-specific coping mechanisms.

Third, this study focuses on a three-month intervention period. Although participant recruitment and data collection occurred between July 2018 and February 2021, this secondary analysis specifically examines the three months during which participants learned and practiced the COBMINDEX intervention. This relatively short timeframe may not have been sufficient time for patients, particularly men, to fully integrate newly learned coping strategies. As a result, the full effects of the intervention may not have fully manifested, and the sustained changes may not have been adequately observed.

Fourth, reliance on self-reported measures introduces the possibility of response bias [125,126,127]. While these tools are standard in psychological research, their subjective nature may have introduced variability in the reliability of our findings. Participants’ interpretations of their experiences, as well as their willingness to report them accurately, may have influenced the data.

Fifth, selection bias may have influenced our findings due to the voluntary nature of participation [127]. Only patients who expressed interest in the COBMINDEX program and a willingness to engage in self-care were included. Participants were informed about the importance of practicing the learned skills at home, which may have influenced their decision to enroll. This self-selection may have resulted in a sample that overrepresents individuals motivated to participate in psychological interventions and commit to regular practice. Furthermore, the gender imbalance, with more women than men agreeing to participate, may reflect broader trends of lower male engagement in psychological interventions, further limiting the generalizability of our findings, particularly to men.

Sixth, the onset of the COVID-19 pandemic in Israel in March 2020 [128] may have impacted data collection, particularly for post-CRP data points. However, the pandemic began nearly two years into the study, after most data had already been collected. Of the 120 participants, 106 completed COBMINDEX before the emergency declaration, five began the intervention before the declaration and completed it afterward, and only nine were recruited post-pandemic onset. Given that fewer than 10% of participants completed the intervention after the pandemic began, the statistical impact of these disruptions on the study’s findings is likely minimal. Nonetheless, as Batran et al. [129] highlight, disruptions in data collection and participant engagement during the pandemic have been widely recognized in health research. While the direct statistical impact appears negligible, broader contextual factors such as quarantine, uncertainty, and health-related concerns may have influenced participants’ experiences and outcomes in ways that remain unclear.

Seventh, the success of blinding among the assessors post-intervention was not formally tested; however, multiple precautions were taken to minimize the potential bias. Gastroenterologists had no access to group assignments, patient files contained no allocation indicators, and laboratory staff received only coded blood samples. Despite these measures, unintentional bias cannot be entirely excluded, and future studies should incorporate formal blinding assessments to enhance methodological rigor and validation [130].

Finally, while minimal changes in participants’ medications during the study did not significantly affect outcomes, the limited scope of medication adjustments may not fully capture the potential influence of pharmacological factors on coping strategies and well-being. Although multiple contextual factors were controlled, unmeasured variables—such as personality traits or the availability of social support—may have also played a role in the relationship between coping strategies and life satisfaction.

### 4.5. Future Research Directions

This study highlights the need for gender-sensitive training in healthcare to reduce disparities in CD management. Future research should refine intervention strategies, extend follow-up periods, and incorporate advanced statistical methods to enhance effectiveness and generalizability.

Tailored interventions should consider demographic differences, such as age, socioeconomic status, and education level. Younger patients may benefit from digital coping tools, such as mobile applications and virtual support groups, while older adults might prefer in-person workshops and traditional therapy. Addressing financial stressors could improve accessibility for lower-income individuals, while adapting educational programs to various literacy levels would enhance engagement and comprehension.

Longer follow-up studies, ideally lasting at least one year, are necessary to determine the long-term impact of COBMINDEX. Extending the study duration would allow patients to internalize cognitive and behavioral strategies, integrate mindfulness practices into their daily routines, and develop more stable, adaptive coping mechanisms. These changes require time to produce measurable psychological and physiological improvements, making extended research crucial for assessing sustained benefits.

Future studies should stratify patients by disease severity and duration and use advanced statistical methods, such as Structural Equation Modeling (SEM), to examine the relationships between coping strategies, psychological factors, and disease progression. This approach would provide deeper insights into how different variables interact and influence patient outcomes.

To further enhance intervention effectiveness, future studies should tailor psychological interventions to gender-specific coping mechanisms. Investigating the role of self-esteem in treatment responses could help identify strategies that strengthen adaptive coping while reducing maladaptive patterns. Additionally, further exploration is needed to understand why psychological stress and life satisfaction appear more strongly correlated in women than in men. Research into whether men experience delayed intervention effects or process stress differently could help refine psychological treatments, ensuring more effective and personalized care for all CD patients.

## 5. Conclusions

This study provides important insights into the effectiveness of the COBMINDEX intervention for managing Crohn’s disease (CD). Both men and women exhibited measurable improvements in coping strategies, with distinct gender-specific differences. Women exhibited greater reliance on emotion-focused and problem-focused coping, with reduced use of dysfunctional coping mechanisms such as self-blame and behavioral disengagement. In contrast, men showed a stronger association between mindfulness and improved life satisfaction, highlighting potential gender-based differences in psychosocial adaptation.

These findings emphasize the importance of integrating structured psychosocial interventions like COBMINDEX into clinical care. Healthcare professionals should integrate COBMINDEX into gastroenterology clinics and IBD support programs. A combination of digital intervention platforms and structured sessions with social workers can provide personalized psychological support and reinforce coping strategies.

A gender-sensitive approach is essential for optimizing psychosocial interventions. Gender-specific assessments using tailored screening tools can help identify differences in coping styles and psychological responses. Interventions should address gender-specific stressors, including hormonal influences in women and societal expectations surrounding emotional expression in men. Comprehensive training for healthcare providers in gender sensitivity and cultural competency is essential to enhance understanding of how gender influences coping and treatment outcomes.

To enhance accessibility and engagement, flexible intervention formats should include tailored educational materials, adjustable scheduling via video conferencing, and separate gender-specific support sessions to foster a more inclusive and supportive environment. Additionally, effective communication strategies—such as active listening and structured follow-ups—should be implemented to ensure ongoing responsiveness to patient needs. A multidisciplinary approach integrating psychologists, gastroenterologists, and social workers can further enhance intervention efficacy. Furthermore, when appropriate, family involvement may provide a holistic support system, reinforcing patient engagement and adherence.

Systematic data collection stratified by gender is essential for monitoring outcomes and refining the COBMINDEX program. Mindfulness-based strategies that consider gender dynamics and safe spaces for emotional expression should be integrated to maximize patient benefit. Empowerment and self-advocacy training, facilitated through skill-building workshops and personalized goal setting, can help patients navigate gender-related barriers in the healthcare system. By implementing these targeted strategies, healthcare professionals can effectively integrate COBMINDEX into clinical practice, ensuring gender-responsive interventions that improve coping mechanisms and quality of life for individuals with CD.

Despite these promising findings, several limitations must be acknowledged. The three-month study duration may have been insufficient to capture the long-term behavioral changes. Furthermore, the unequal gender distribution, with fewer male participants, may have affected the ability to fully assess gender-based intervention effects. Future studies should aim for longer intervention periods and more balanced samples to further evaluate the sustained benefits of COBMINDEX.

While short-term interventions like COBMINDEX have demonstrated positive effects, sustained improvements likely depend on ongoing education, structured follow-ups, and continued adaptation of intervention strategies. By implementing these recommendations, healthcare professionals can effectively integrate COBMINDEX into routine care, ensuring long-term psychological well-being and improved patient outcomes.

## Figures and Tables

**Table 1:** Gender-based comparative analysis of cohort characteristics at study enrollment.

Characteristics	Men n = 45 (37.5%)	Women n = 75 (62.5%)
Age, median [IQR], years	30 [26–39]	31 [26–43.25]
Married/paired, n [%]	22 (49)	36 (48)
Education level, n [%]		
High school or vocational studies	11 (24)	16 (21)
College or university	34 (76)	59 (79)
Education, median [IQR], years	14 [12–16]	15 [12–16]
Economic status, n [%]		
Low	16 (36)	14 (19)
Middle-high	29 (64)	61 (81)
Current employment, n [%]	35 (78)	53 (71)
Current smoker, n [%]	4 (9)	9 (12)
Illness duration, median [IQR], years	7.1 [4–13.8] *	4.9 [2.4–12.3]
BMI, median [IQR],	21.8 [20.4–24.8]	22.3 [19.5–25.5]
Current medication, n [%]		
Steroids	3 (7)	3 (4)
Immunomodulators	13 (29)	7 (9)
Biologics	26 (58)	27 (36)
Montreal classification, n [%] Age at diagnosis, y		
A1: ≤16	4 (9)	8 (11)
A2: 17–40	41 (91)	61 (81)
A3: ≥40	0	6 (8)
Location		
L1: Ileal	31 (69)	38 (51)
L2: Colonic	2 (4)	9 (12)
L3: Ileocolonic	11 (24)	27 (36)
L4: Isolated upper disease	1 (2)	1 (1)
Behavior		
B1: Non-stricturing, non-penetrating	24 (53)	49 (65)
B2: Stricturing	18 (40)	20 (27)
B3: Penetrating	4 (9)	9 (9)
Perianal	10 (22)	11 (25)
Extraintestinal manifestations, n [%]	28 (62)	51 (68)

* *p* < 0.05.

**Table 2:** (**a**) Pre- and post-COBMINDEX gender differences in clinical, biological, and psychological aspects: a comparative analysis within and between genders. (**b**) Pre- and post-COBMINDEX gender differences in mindfulness and coping strategies: a comparative analysis within and between genders.

(a)								
	Pre-COBMINDEX			Post-COBMINDEX			*p* Within Genders	
	Men	Women	*p* Between Genders	Men	Women	*p* Between Genders	Men	Women
	Median (Min–Max) [IQR]			Median (Min–Max) [IQR]				
HBI	7	7	NS	3	4	NS	<0.001	<0.001
	(2–15) [6–10]	(1–15) [6–9]		(0–17) [2–6]	(0–18) [2–6]			
CRP	0.49	0.5	NS	0.4	0.3	NS	NS	NS
	(0–15) [0.3–1.1]	(0–6) [0.1–1.1]		(0–11) [0.2–1.3]	(0–4.1) [0.1–0.8]			
Satisfaction With Life	20	21	NS	23	24	NS	0.006	0.001
	(7–32) [13–24]	(9–35) [16–26]		(5–35) [16.5–28]	(10–34) [19–28]			
Global Severity Index	1.0	0.98	NS	0.79	0.72	NS	<0.001	<0.001
	(0.06–2.3) [0.7–1.7]	(0.02–3.0) [0.6–1.5]		(0.08–2.3) [0.4–1.3]	(0.06–2.4) [0.45–1.3]			
(**b**)								
	**Pre-COBMINDEX**			**Post-COBMINDEX**			***p* Within** **Genders**	
	**Men**	**Women**	***p*** **Between Genders**	**Men**	**Women**	***p*** **Between Genders**	**Men**	**Women**
	**Median** **(Min–Max) [IQR]**			**Median** **(Min–Max) [IQR]**				
**Mindfulness**	34	32	NS	37	37	NS	0.035	<0.001
	(17–51) [28.5–40]	(21–50) [28–39]		(19–56) [30.5–42]	(24–52) [34–42]			
**Emotion-Focused Coping**	25	25	NS	27	29	NS	<0.001	<0.001
	(17–38) [22–28]	(14–38) [23–28]		(19–37) [24–30.5]	(8–38) [25–32]			
Emotional support	5	5	NS	5	6	0.010	NS	0.001
	(2–8) [3.5–6]	(2–8) [4–6]		(2–8) [4–6]	(2–8) [4–7]			
Positive reframing	5	6	NS	6	6	NS	0.003	0.001
	(2–8) [4–6]	(1–8) [4–7]		(2–8) [4–7]	(3–8) [5–7]			
Humor	5	5	NS	6	5	NS	NS	NS
	(2–8) [3–6]	(2–8) [3.8–6]		(2–8) [4–7]	(2–8) [4–6]			
Acceptance	7	7	NS	7	7	NS	NS	NS
	(4–8) [6–8]	(4–8) [6–8]		(4–8) [6–8]	(4–8) [7–8]			
Religion	3	3	NS	5	5	NS	<0.001	<0.001
	(2–8) [2–4.5]	(2–8) [2–5]		(2–8) [4–7]	(2–8) [4–6]			
**Problem-Focused Coping**	17	17	NS	18	19	NS	NS	0.014
	(9–24) [15–19]	(11–24) [15–20]		(9–24) [15.5–20.5]	(8–24) [17–21]			
Active coping	6	6	NS	7	7	NS	0.034	0.027
	(3–8) [5–7]	(3–8) [5–8]		(4–8) [6–8]	(4–8) [6–8]			
Planning	6	6	NS	7	7	NS	NS	NS
	(2–8) [5.5–7.5]	(2–8) [5–7]		(3–8) [5–8]	(2–8) [5–7]			
Instrumental Support	4	5	0.044	5	6	0.010	NS	NS
	(2–8) [3.5–6]	(2–8) [4–6]		(2–8) [4–6]	(1–8) [4–7]			
**Dysfunctional Coping**	20	19	NS	20	18	0.023	NS	0.016
	(12–29) [17–24]	(10–27) [16–22]		(14–24) [17–21.5]	(10–27) [16–20]			
Behavioral disengage	2	2	NS	2	2	NS	NS	0.011
	(2–6) [2–4]	(2–8) [2–4]		(2–8) [2–4]	(2–6) [2–3]			
Self-blame	5	5	NS	5	5	NS	NS	0.003
	(3–8) [4–7]	(2–8) [4–7]		(3–8) [4–6.5]	(2–8) [4–5]			
Denial	2	2	NS	2	2	NS	NS	NS
	(2–8) [2–4	(2–8) [2–3]		(2–8) [2–3]	(2–8) [2–3]			
Substance use	2	2	NS	2	2	NS	NS	NS
	(2–8) [2–4]	(2–8) [2–3]		(2–8) [2–4]	(2–8) [2–4]			
Self-distraction	6	6	NS	6	6	NS	NS	NS
	(2–8) [5–7]	(2–8) [4–7]		(2–8) [5–7]	(2–8) [4–7]			

N Men = 45; N Women = 75.

**Table 3:** Quantile regression analysis: comparing determinants of life satisfaction in men and women.

	Independent Variables	Regression Coefficient *(p)*				
		10%	25%	50%	75%	90%
Men	Emotion-focused coping	0.44 ***	0.43	0.30	0.12	1.36
	Problem-focused coping	0.04	0.16	0.05	0.58	−0.50
	Dysfunctional coping	−0.57 ***	−0.65 *	−0.37	−1.23	−1.19
	Mindfulness	0.10	0.51 *	0.53 *	0.60	0.15
	Global Severity Index (GSI)	0.11	0.10	−0.17	−0.00	−0.03
Women	Emotion-focused coping	0.21	0.06	−0.04	−0.21	−0.38
	Problem-focused coping	−0.00	0.06	0.22	0.37	0.14
	Dysfunctional coping	−0.13	−0.17	−0.36 *	−0.81 *	−0.39
	Mindfulness	0.04	0.18	0.29	0.01	−0.22
	Global Severity Index (GSI)	−0.18 ***	−0.14 **	−0.15 **	0.01	−0.04

* *p* < 0.05, ** *p* < 0.01, *** *p* < 0.001.

## Data Availability

The dataset of this study is not publicly available, owing to the sensitive nature of the patient data and the rules of the Ethics Committees. However, the corresponding author is willing to consider requests from interested parties.
